# Interactive Effects of Situational Variables Regarding Teams’ Technical Performance in the UEFA Champions League

**DOI:** 10.3389/fpsyg.2022.781376

**Published:** 2022-03-17

**Authors:** Qing Yi, Jingyong Yang, Xinlei Wang, Yang Gai, Miguel-Ángel Gómez-Ruano

**Affiliations:** ^1^School of Physical Education and Sport Training, Shanghai University of Sport, Shanghai, China; ^2^Shanghai Key Lab of Human Performance, Shanghai University of Sport, Shanghai, China; ^3^International College of Football, Tongji University, Shanghai, China; ^4^School of Physical Education, Ningbo University, Ningbo, China; ^5^Football Club Sochaux-Montbéliard, Montbéliard, France; ^6^Facultad de Ciencias de la Actividad Física y del Deporte (INEF), Universidad Politécnica de Madrid, Madrid, Spain

**Keywords:** football, soccer, situational variable, performance indicator, match analysis

## Abstract

The aim of this study was to examine the interactive effects of situational variables (competition stage, match location, and match outcome) on teams’ technical performance in the UEFA Champion League. Match data of 19 technical actions and events were collected and classified into three groups (variables related to goal scoring, offense, and defense) during eight seasons (2009/2010–2016/2017). Repeated-measures analysis of variance (RMANOVA) was used to identify the differences in the technical performances among teams. Results showed that the significant differences in the technical performance between the group stage and the knockout stage were only found in dribble and yellow card. However, differences in the variables related to goal scoring and offense were more significant than in the variables related to defense under the comparisons across competition situations, and the differences in the variables related to defense were mainly detected in the yellow card. The number of variables that showed significant differences among match location and match outcome in the group stage were less than in the knockout stage. Therefore, the identified key performance indicators by considering the interactive effects of situational variables may provide detailed and practical insights for coaches to develop useful training interventions and match strategies for upcoming match playing in specific competition situations.

## Introduction

Football is a complex team sport where the match performance includes the interactions of technical, tactical, and physical factors under different competition situations, which can be captured using semi-automated computerized tracking systems during match play (Gai et al., 2019). Previous studies have identified that teams’ technical match performance can be influenced by situational variables such as match location (playing at home or away), match outcome (win, draw, or lose), and/or competition stage (group stage or knockout stage) (Page and Page, 2007; Taylor et al., 2010; Lago-Peñas, 2012; Gómez et al., 2013). In particular, Lago-Peñas and Lago-Ballesteros (2011) reported that teams’ technical performance indicators, such as the number of shots and ball possession, showed significant differences between home and away matches in the Spanish professional men’s league. Pollard (2008) found that the home environment (e.g., the social support of the crowd) is associated with an increased functional aggressive response manifested by more offensive behaviors. Taylor et al. (2008) indicated that match outcome is determined by whether a team is winning, drawing, or losing at the time a particular behavior is recorded. Specifically, Lago and Martín (2007) reported that the importance of match outcome is reflected in changes in team strategy in response to the score line. Furthermore, teams’ match performance may also differ among competition stages (e.g., group stage and knockout stage), but this issue has been less thoroughly discussed in previous research. Only a recent study from Yi et al. (2020b)- investigated the effects of competition stages on players’ technical performance in the UEFA Champions League, concluding that there were no differences in variables related to goal scoring, attacking, passing, and defending between five playing positions (central defender, full back, central midfielder, wide midfielder, and forward) from group and knockout stages, except for fouls.

Technical indicators have been considered as better factors compared to physical indicators to predict a team’s success (Clemente et al., 2016). Given the aforementioned effects of situational variables, researchers have attempted to establish the technical performance profiles considering the situational variables in football (O’Donoghue, 2013; Liu et al., 2015b; Liu et al., 2016a). Technical performance profiles may provide a useful tool for coaches and analysts to evaluate the match performance at both team and player level. Yang et al. (2018) reported that teams in the top-ranked group exhibited higher OPP (percentage of total match time with possession in opponent’s half), final 1/3 (number of entry passes in the final 1/3 of the field), penalty area entries, and 50–50 challenges [percentage of even (50–50)] challenges won compared to teams in the lower–middle-ranked groups in the Chinese Super League. Similarly, Hughes and Franks (2005) reported that the converting possessions into shots on goal was an important factor for success; successful teams had higher conversion rates than unsuccessful teams in the 1990 FIFA World Cup. Hook and Hughes (2001) demonstrated that successful teams exhibited a greater match time with ball possession than unsuccessful teams in the UEFA associations (English Premier League, Spanish La Liga, German Bundesliga, Italian Serie A, French Ligue 1, and the other European associations).

Even though the above mentioned studies conducted detailed assessments of the effects of each situational variable on the technical performance of teams and players (Taylor et al., 2010; Lago-Peñas and Lago-Ballesteros, 2011; Liu et al., 2015b; Yi et al., 2020b), but they did not account for considering the possibility of higher-order interactions across competition scenarios by analyzing the effects of competition contexts on the technical performance under other ones. The examination of situational variables in isolation would appear to provide limited insight into the complex nature of football match performance (McGarry and Franks, 2003), a limited sample size may be one of the main factors restricting the assessment of interactive effects. Therefore, the current study aimed to investigate the differences of teams’ technical performance regarding interactive effects across situational variables (match outcome, match location, and competition stage) in the UEFA Champions League based on a database of eight seasons (from 2009/10 to 2016/17).

## Materials and Methods

### Sample and Variables

The sample consisted of 768 matches (2,000 observations) in the UEFA Champions League from the 2009/10 to 2016/17 seasons. Data were collected from a public-access football statistics website named “whoscored.com.”^1^ The original data of the website was provided by OPTA Sportsdata. The reliability of the technical data from the tracking system (OPTA Client System) was previously verified (intra-class correlation coefficient: 0.88–1.00; standardized typical error: 0.00–0.37) (Liu et al., 2013). This study was conducted in accordance with the Declaration of Helsinki, and the local Institutional Review Board approved the study.

Nineteen technical performance variables were divided into three categories (variables related to goal scoring, offense, and defense), and the operational definitions were defined previously (Yi et al., 2019b). The following variables were examined. (1) Variables related to goal scoring: shot, shot on target (ShotOT), shot from open play (ShotOP), shot from set piece (ShotSP), and shot from counter-attack (ShotCA). (2) Variables related to offense: possession, pass, pass success (PS), short ball (SB), long ball (LB), cross, through ball (TB), dribble, aerial success (AS), corner, offside. (3) Variables related to defense: tackle, foul, and yellow card (YC).

### Statistical Analysis

After testing the data to determine whether it followed a normal distribution (Kolmogorov–Smirnov), we used repeated-measures analysis of variance (RMANOVA) to identify the differences in teams’ technical performance across competition stages, match locations, and match outcomes; pairwise comparisons were performed with Tukey correction. All statistical analyses were conducted using IBM SPSS software for Windows Version 21.0 (IBM Corp, Armonk, NY, United States), and the level of significance was set at 0.05. Season, competition stage, match location, and match outcome were included as the repeated-measure factors to account for repeated measurement on the teams, as well as the number of matches each team played across different seasons, competition stages, match locations, and match outcomes. Estimated effect sizes and their 90% confidence limits were expressed in standardized units, and the magnitudes of effect size were evaluated qualitatively using the scale initially suggested by Cohen and expanded by Sawilowsky: 0.01 = very small, 0.2 = small, 0.5 = medium, 0.8 = large, 1.2 = very large, and 2.0 = huge (Sawilowsky, 2009).

## Results

### Effects of Competition Stages

Table 1 presents the differences in teams’ technical performance between the group and knockout stages in the UEFA Champions League. No significant differences were observed in all variables between the group stage and knockout stage, except for the variables dribble (ES: −0.31 ± 0.09) and yellow card (ES: −0.26 ± 0.09).

**TABLE 1:** Comparison of teams’ technical match statistics between group stage and knockout stage in the UEFA Champions League.

Variable	Group stage	Knockout stage	Effect size
	(M ± SD)	(M ± SD)	(ES ± 90%CI)
Shot	13.39 ± 6.06	13.04 ± 5.86	0.06 ± 0.09
ShotOT	4.77 ± 2.90	4.74 ± 2.64	0.01 ± 0.09
ShotOP	9.03 ± 4.67	8.81 ± 4.64	0.05 ± 0.09
ShotSP	3.02 ± 2.12	3.05 ± 2.12	−0.01 ± 0.09
ShotCA	0.43 ± 0.76	0.38 ± 0.70	0.08 ± 0.09
Possession	50.00 ± 11.97	50.00 ± 13.39	0.00 ± 0.09
Pass	498.03 ± 135.93	504.26 ± 148.04	−0.04 ± 0.09
PS	80.09 ± 7.03	79.82 ± 7.72	0.04 ± 0.09
SB	414.71 ± 131.75	423.04 ± 142.95	−0.06 ± 0.09
LB	61.36 ± 13.57	59.42 ± 13.96	0.14 ± 0.09
Cross	19.60 ± 11.71	18.67 ± 8.71	0.09 ± 0.10
TB	2.55 ± 2.79	3.13 ± 3.68	−0.18 ± 0.09
Dribble	8.77 ± 4.63	10.29 ± 5.19	−0.31 ± 0.09*
AS	50.01 ± 14.28	50.01 ± 13.46	0.00 ± 0.09
Corner	5.06 ± 2.98	4.90 ± 2.86	0.05 ± 0.09
Offside	2.60 ± 2.09	2.46 ± 2.01	0.07 ± 0.09
Tackle	20.20 ± 5.96	20.96 ± 6.15	−0.12 ± 0.09
Foul	13.46 ± 4.40	13.92 ± 4.72	−0.10 ± 0.09
YC	1.84 ± 1.26	2.20 ± 1.41	−0.26 ± 0.09*

*Descriptive statistics of teams are presented as mean ± standard deviation (M ± SD). Effect sizes (ES) are presented as the magnitude of the true difference in means ± 90% confidence interval. Asterisks indicate a p-value lower than the significance level of 0.05. ShotOT, shot on target; ShotOP, shot from open play; ShotSP, shot from set piece; ShotCA, shot from counter-attack; PS, pass success; SB, short ball; LB, long ball; TB, through ball; AS, aerial success; YC, yellow card.*

### Effects of Match Locations and Competition Stages on Match Outcome

Tables 2, 3 present the differences in technical performance between winning, drawing, and losing teams played at different locations in the group stage and knockout stage, respectively. Significant differences were found in most of the variables related to goal scoring, offense, and defense across match outcomes when played at home in the group stage (ES: −0.42 ± 0.17 to 1.01 ± 0.15), with the exception of the comparisons between drawing and losing matches in variables related to defense. Significant differences in variables related to goal scoring and offense (ES: 0.24 ± 0.18 to 1.02 ± 0.15) and non-significant differences in variables related to defense were observed among three match results when played away in the group stage. Conversely, in comparison with the group stage, the number of variables that showed obvious differences across match outcomes were less when played at both home and away matches in the knockout stage. In the comparisons of winning vs. drawing and winning vs. losing, only shot, shot on target, shot from open play, shot from counter-attack, possession, and offside showed significant differences in home matches (ES: 0.41 ± 0.31 to 0.88 ± 0.29). In addition to these variables, pass, pass success, short ball, and yellow card also showed significant differences in away matches (ES: 0.41 ± 0.34 to 1.01 ± 0.29). No significant differences were observed in any of the three types of variables between drawing and losing matches when played at home and away.

**TABLE 2:** Comparisons of technical match statistics between winning (W), drawing (D), and losing (L) teams playing at different locations in the group stage of the UEFA Champions League.

Variable	Home						Away					
		
	Win	Draw	Lose	*Post-hoc* (ES ± 90%CI)			Win	Draw	Lose	*Post-hoc* (ES ± 90%CI)		
								
	(M ± SD)	(M ± SD)	(M ± SD)	W-D	W-L	D-L	(M ± SD)	(M ± SD)	(M ± SD)	W-D	W-L	D-L
Shot	16.87 ± 6.06	14.53 ± 5.96	12.63 ± 5.70	0.38 ± 0.18*	0.68 ± 0.16*	0.32 ± 0.21*	13.69 ± 5.40	12.38 ± 5.76	10.12 ± 4.73	0.24 ± 0.19*	0.68 ± 0.17*	0.44 ± 0.19*
ShotOT	6.83 ± 2.96	4.76 ± 2.55	3.66 ± 2.26	0.69 ± 0.17*	1.01 ± 0.15*	0.45 ± 0.23*	5.79 ± 2.87	4.23 ± 2.41	3.03 ± 1.93	0.56 ± 0.17*	1.02 ± 0.15*	0.55 ± 0.20*
ShotOP	11.47 ± 4.90	9.72 ± 4.73	8.39 ± 4.13	0.36 ± 0.18*	0.63 ± 0.17*	0.30 ± 0.22*	9.12 ± 4.41	8.52 ± 4.23	6.83 ± 3.80	0.14 ± 0.19	0.54 ± 0.17*	0.42 ± 0.19*
ShotSP	3.63 ± 2.15	3.51 ± 2.39	2.98 ± 2.07	0.05 ± 0.19	0.30 ± 0.18*	0.24 ± 0.20*	3.00 ± 1.82	2.72 ± 2.35	2.37 ± 1.79	0.13 ± 0.19	0.34 ± 0.18*	0.17 ± 0.18
ShotCA	0.63 ± 0.94	0.36 ± 0.70	0.23 ± 0.54	0.30 ± 0.17*	0.48 ± 0.16*	0.21 ± 0.26	0.59 ± 0.80	0.47 ± 0.72	0.29 ± 0.61	0.17 ± 0.19	0.43 ± 0.17*	0.27 ± 0.20*
Possession	54.39 ± 11.59	51.54 ± 10.74	46.45 ± 11.63	0.25 ± 0.19*	0.65 ± 0.16*	0.44 ± 0.20*	53.55 ± 11.63	48.46 ± 10.74	45.61 ± 11.59	0.44 ± 0.20*	0.65 ± 0.16*	0.25 ± 0.19*
Pass	547.35 ± 150.87	503.33 ± 116.59	462.21 ± 109.45	0.31 ± 0.18*	0.60 ± 0.16*	0.36 ± 0.24*	543.87 ± 154.43	473.36 ± 120.96	451.77 ± 111.06	0.49 ± 0.18*	0.67 ± 0.16*	0.19 ± 0.21
PS	82.28 ± 7.27	79.93 ± 6.60	78.86 ± 6.51	0.33 ± 0.18*	0.48 ± 0.17*	0.16 ± 0.22	81.56 ± 6.96	78.56 ± 7.45	78.55 ± 6.41	0.41 ± 0.19*	0.44 ± 0.17*	0.003 ± 0.19
SB	461.46 ± 149.19	415.98 ± 113.37	378.71 ± 101.93	0.32 ± 0.17*	0.60 ± 0.16*	0.34 ± 0.24*	459.49 ± 151.94	390.25 ± 116.42	373.66 ± 106.00	0.49 ± 0.18*	0.65 ± 0.16*	0.15 ± 0.21
LB	61.27 ± 13.71	62.26 ± 14.19	59.79 ± 13.29	−0.07 ± 0.19	0.11 ± 0.18	0.18 ± 0.21	63.54 ± 13.84	63.09 ± 13.62	59.81 ± 12.84	0.03 ± 0.20	0.28 ± 0.17*	0.25 ± 0.19*
Cross	21.33 ± 8.43	22.61 ± 9.32	21.99 ± 10.03	−0.15 ± 0.20	−0.07 ± 0.17	0.06 ± 0.19	18.59 ± 20.76	17.57 ± 8.98	16.52 ± 8.48	0.06 ± 0.17	0.14 ± 0.16	0.12 ± 0.29
TB	3.29 ± 3.27	2.55 ± 2.93	1.72 ± 1.95	0.23 ± 0.17*	0.53 ± 0.17*	0.34 ± 0.24*	3.45 ± 3.20	2.44 ± 2.66	1.81 ± 1.96	0.33 ± 0.17*	0.62 ± 0.17*	0.28 ± 0.22*
Dribble	10.13 ± 5.17	8.45 ± 4.09	7.98 ± 4.43	0.34 ± 0.18*	0.43 ± 0.16*	0.11 ± 0.23	9.40 ± 4.37	8.33 ± 4.55	7.88 ± 4.28	0.24 ± 0.20*	0.35 ± 0.17*	0.10 ± 0.19
AS	54.59 ± 14.25	51.12 ± 13.94	46.90 ± 13.09	0.24 ± 0.18*	0.54 ± 0.17*	0.31 ± 0.21*	53.13 ± 13.07	48.89 ± 13.95	45.45 ± 14.23	0.31 ± 0.21*	0.54 ± 0.17*	0.24 ± 0.18*
Corner	5.84 ± 2.87	5.80 ± 3.10	5.27 ± 3.15	0.02 ± 0.19	0.19 ± 0.17	0.17 ± 0.20	4.85 ± 2.79	4.63 ± 2.86	4.13 ± 2.80	0.08 ± 0.21	0.19 ± 0.17	0.18 ± 0.19
Offside	2.89 ± 2.31	2.78 ± 2.13	2.73 ± 2.10	0.05 ± 0.19	0.07 ± 0.17	0.02 ± 0.22	2.82 ± 1.92	2.24 ± 2.02	2.19 ± 1.90	0.29 ± 0.20*	0.33 ± 0.17*	0.03 ± 0.19
Tackle	20.55 ± 6.09	20.19 ± 5.79	19.57 ± 6.22	0.06 ± 0.19	0.16 ± 0.17	0.10 ± 0.21	20.18 ± 5.77	20.52 ± 5.79	20.12 ± 5.95	−0.06 ± 0.21	0.01 ± 0.17	0.07 ± 0.19
Foul	12.80 ± 4.25	13.18 ± 4.06	13.67 ± 4.16	−0.09 ± 0.19	−0.21 ± 0.17*	−0.12 ± 0.21	13.48 ± 4.51	13.81 ± 4.42	13.95 ± 4.69	−0.07 ± 0.21	−0.10 ± 0.17	−0.03 ± 0.19
YC	1.37 ± 1.13	1.76 ± 1.23	1.90 ± 1.34	−0.32 ± 0.20*	−0.42 ± 0.17*	−0.11 ± 0.19	1.92 ± 1.28	2.05 ± 1.10	2.17 ± 1.29	−0.11 ± 0.21	−0.19 ± 0.17	−0.09 ± 0.19

*Descriptive statistics of teams in Tables 2–5 are presented as mean ± standard deviation (M ± SD). Effect sizes (ES) are presented as the magnitude of the true difference in means ± 90% confidence interval. Asterisks indicate that the p-value is lower than the significance level of 0.05. Abbreviations: ShotOT, shot on target; ShotOP, shot from open play; ShotSP, shot from set piece; ShotCA, shot from counter-attack; PS, pass success; SB, short ball; LB, long ball; TB, through ball; AS, aerial success; YC, yellow card.*

**TABLE 3:** Comparisons of technical match statistics between winning, drawing, and losing teams playing at different locations in the knockout stage of the UEFA Champions League.

Variable	Home						Away					
		
	Win	Draw	Lose	*Post-hoc* (ES ± 90%CI)			Win	Draw	Lose	*Post-hoc* (ES ± 90%CI)		
								
	(M ± SD)	(M ± SD)	(M ± SD)	W-D	W-L	D-L	(M ± SD)	(M ± SD)	(M ± SD)	W-D	W-L	D-L
Shot	15.88 ± 5.79	13.50 ± 7.10	12.86 ± 5.05	0.38 ± 0.35	0.53 ± 0.34*	0.11 ± 0.41	13.97 ± 4.88	11.10 ± 4.65	10.37 ± 5.21	0.58 ± 0.42*	0.67 ± 0.31*	0.14 ± 0.37
ShotOT	6.50 ± 2.59	4.02 ± 2.10	4.12 ± 2.23	0.92 ± 0.33*	0.88 ± 0.29*	−0.04 ± 0.47	5.78 ± 2.50	4.05 ± 2.24	3.27 ± 2.01	0.68 ± 0.36*	1.01 ± 0.29*	0.37 ± 0.39
ShotOP	10.69 ± 4.78	9.17 ± 5.48	8.77 ± 3.88	0.31 ± 0.35	0.42 ± 0.34*	0.09 ± 0.43	9.60 ± 4.32	7.45 ± 3.66	6.94 ± 4.15	0.51 ± 0.41*	0.61 ± 0.31*	0.13 ± 0.38
ShotSP	3.75 ± 2.31	3.43 ± 2.50	3.35 ± 2.14	0.14 ± 0.36	0.18 ± 0.34	0.03 ± 0.42	2.67 ± 1.85	2.55 ± 1.68	2.44 ± 1.75	0.07 ± 0.42	0.13 ± 0.33	0.06 ± 0.38
ShotCA	0.56 ± 0.83	0.24 ± 0.48	0.00 ± 0.00	0.41 ± 0.31*	0.76 ± 0.29*	0.71 ± 0.82	0.67 ± 0.99	0.31 ± 0.60	0.31 ± 0.56	0.41 ± 0.34*	0.49 ± 0.31*	0.01 ± 0.45
Possession	52.52 ± 13.57	49.43 ± 13.87	46.69 ± 11.93	0.23 ± 0.36	0.44 ± 0.33*	0.21 ± 0.43	53.31 ± 11.93	50.57 ± 13.87	47.48 ± 13.57	0.21 ± 0.43	0.44 ± 0.33*	0.23 ± 0.36
Pass	523.17 ± 154.54	503.52 ± 146.65	477.22 ± 113.48	0.13 ± 0.35	0.32 ± 0.33	0.20 ± 0.47	555.76 ± 162.47	513.98 ± 149.48	470.85 ± 140.83	0.26 ± 0.39	0.56 ± 0.32*	0.30 ± 0.38
PS	80.69 ± 8.09	79.43 ± 6.70	79.97 ± 5.90	0.16 ± 0.35	0.10 ± 0.32	−0.09 ± 0.49	82.52 ± 7.00	79.26 ± 7.82	77.96 ± 8.31	0.43 ± 0.44	0.56 ± 0.32*	0.16 ± 0.36
SB	439.76 ± 150.16	418.79 ± 141.82	397.86 ± 108.27	0.14 ± 0.35	0.30 ± 0.33	0.17 ± 0.47	474.48 ± 160.37	431.69 ± 139.79	392.54 ± 135.44	0.28 ± 0.39	0.55 ± 0.32*	0.29 ± 0.38
LB	59.73 ± 13.82	58.81 ± 14.11	55.95 ± 12.38	0.07 ± 0.36	0.28 ± 0.33	0.22 ± 0.43	61.28 ± 13.64	62.74 ± 17.11	58.97 ± 13.61	−0.10 ± 0.40	0.17 ± 0.35	0.26 ± 0.36
Cross	19.92 ± 8.14	22.74 ± 10.63	20.95 ± 9.12	−0.32 ± 0.37	−0.12 ± 0.35	0.18 ± 0.38	16.48 ± 7.78	16.07 ± 8.32	16.88 ± 8.03	0.05 ± 0.42	−0.05 ± 0.33	−0.10 ± 0.37
TB	3.77 ± 4.32	3.60 ± 5.12	2.47 ± 2.22	0.04 ± 0.33	0.34 ± 0.35	0.30 ± 0.45	3.52 ± 3.89	3.12 ± 2.93	2.47 ± 2.91	0.11 ± 0.38	0.32 ± 0.32	0.22 ± 0.40
Dribble	10.99 ± 5.41	11.40 ± 5.21	10.91 ± 5.41	−0.08 ± 0.37	0.01 ± 0.33	0.09 ± 0.42	10.43 ± 5.24	10.29 ± 4.31	8.84 ± 4.89	0.03 ± 0.42	0.32 ± 0.32	0.30 ± 0.38
AS	52.39 ± 13.59	47.83 ± 14.32	48.88 ± 12.11	0.33 ± 0.35	0.27 ± 0.33	−0.08 ± 0.43	51.16 ± 12.11	52.17 ± 14.32	47.64 ± 13.60	−0.08 ± 0.43	0.27 ± 0.33	0.33 ± 0.35
Corner	5.68 ± 2.90	5.19 ± 3.26	5.14 ± 2.85	0.16 ± 0.36	0.19 ± 0.34	0.02 ± 0.41	4.40 ± 2.43	4.69 ± 2.87	4.23 ± 2.70	−0.11 ± 0.43	0.07 ± 0.33	0.17 ± 0.36
Offside	2.85 ± 2.19	2.26 ± 1.99	2.00 ± 1.73	0.27 ± 0.35	0.41 ± 0.32*	0.14 ± 0.47	2.83 ± 1.89	2.45 ± 1.85	2.17 ± 1.99	0.20 ± 0.43	0.33 ± 0.32	0.14 ± 0.37
Tackle	21.26 ± 6.46	20.81 ± 5.93	19.50 ± 5.58	0.07 ± 0.36	0.28 ± 0.32	0.23 ± 0.45	21.22 ± 5.46	21.33 ± 6.73	21.13 ± 6.27	−0.02 ± 0.43	0.02 ± 0.34	0.03 ± 0.36
Foul	13.52 ± 4.75	13.24 ± 4.38	13.33 ± 3.58	0.06 ± 0.35	0.04 ± 0.33	−0.02 ± 0.47	13.69 ± 4.95	14.29 ± 5.06	14.80 ± 4.98	−0.12 ± 0.42	−0.22 ± 0.33	−0.10 ± 0.37
YC	1.93 ± 1.37	1.86 ± 1.30	2.00 ± 1.11	0.05 ± 0.35	−0.06 ± 0.33	−0.12 ± 0.46	1.98 ± 1.37	2.57 ± 1.50	2.65 ± 1.48	−0.41 ± 0.42	−0.45 ± 0.32*	−0.05 ± 0.36

*Descriptive statistics of teams in Tables 2–5 are presented as mean ± standard deviation (M ± SD). Effect sizes (ES) are presented as the magnitude of the true difference in means ± 90% confidence interval. Asterisks indicate that the p-value is lower than the significance level of 0.05. Abbreviations: ShotOT, shot on target; ShotOP, shot from open play; ShotSP, shot from set piece; ShotCA, shot from counter-attack; PS, pass success; SB, short ball; LB, long ball; TB, through ball; AS, aerial success; YC, yellow card.*

### Effects of Match Outcome and Competition Stage on Match Location

Tables 4, 5 present the differences in technical performance between home and away matches considering match outcomes in the group stage and knockout stage, respectively. In the group stage, a similar trend was found, where the teams that played at home showed a higher number of shots, shots on target, shots from open play, shots from set piece, and corners (ES: 0.21 ± 0.18 to 0.58 ± 0.14), and fewer yellow cards (ES: −0.45 ± 0.14 to −0.20 ± 0.14) than the teams that played away throughout all three types of match results. No significant differences were detected in variables related to passing between match locations regarding winning matches. In contrast, there were fewer variables that showed significant differences in the knockout stage, especially for drawing matches—only cross (ES: 0.66 ± 0.34) and yellow card (ES: −0.60 ± 0.35) presented significant differences. There were no significant differences observed between home and away matches in variables related to defense when considering the match outcome of win, and in variables related to goal scoring when considering the match outcome of draw, but the differences in all the variables related to goal scoring between home and away matches when considering the match outcome of lose were significant (ES: -0.63 ± 0.17 to 0.47 ± 0.26).

**TABLE 4:** Comparisons of technical match statistics between home and away teams considering match outcomes in the group stage of the UEFA Champions League.

Variable	Win			Draw			Lose		
	Home	Away	Effect size	Home	Away	Effect size	Home	Away	Effect size
	(M ± SD)	(M ± SD)	(ES ± 90%CI)	(M ± SD)	(M ± SD)	(ES ± 90%CI)	(M ± SD)	(M ± SD)	(ES ± 90%CI)
Shot	16.87 ± 6.06	13.69 ± 5.40	0.53 ± 0.13*	14.53 ± 5.96	12.38 ± 5.76	0.36 ± 0.17*	12.63 ± 5.70	10.12 ± 4.73	0.48 ± 0.14*
ShotOT	6.83 ± 2.96	5.79 ± 2.87	0.35 ± 0.14*	4.76 ± 2.55	4.23 ± 2.41	0.21 ± 0.18*	3.66 ± 2.26	3.03 ± 1.93	0.30 ± 0.14*
ShotOP	11.47 ± 4.90	9.12 ± 4.41	0.48 ± 0.14*	9.72 ± 4.73	8.52 ± 4.23	0.26 ± 0.17*	8.39 ± 4.13	6.83 ± 3.80	0.39 ± 0.14*
ShotSP	3.63 ± 2.15	3.00 ± 1.82	0.31 ± 0.13*	3.51 ± 2.39	2.72 ± 2.35	0.33 ± 0.17*	2.98 ± 2.07	2.37 ± 1.79	0.32 ± 0.14*
ShotCA	0.63 ± 0.94	0.59 ± 0.80	0.04 ± 0.14	0.36 ± 0.70	0.47 ± 0.72	−0.14 ± 0.18	0.23 ± 0.54	0.29 ± 0.61	−0.10 ± 0.14
Possession	54.39 ± 11.59	53.55 ± 11.63	0.07 ± 0.14	51.54 ± 10.74	48.46 ± 10.74	0.28 ± 0.17*	46.45 ± 11.63	45.61 ± 11.59	0.07 ± 0.14
Pass	547.35 ± 150.87	543.87 ± 154.43	0.02 ± 0.14	503.33 ± 116.59	473.36 ± 120.96	0.25 ± 0.17*	462.21 ± 109.45	451.77 ± 111.06	0.09 ± 0.14
PS	82.28 ± 7.27	81.56 ± 6.96	0.10 ± 0.14	79.93 ± 6.60	78.56 ± 7.45	0.19 ± 0.18	78.86 ± 6.51	78.55 ± 6.41	0.05 ± 0.14
SB	461.46 ± 149.19	459.49 ± 151.94	0.01 ± 0.14	415.98 ± 113.37	390.25 ± 116.42	0.22 ± 0.17*	378.71 ± 101.93	373.66 ± 106.00	0.05 ± 0.14
LB	61.27 ± 13.71	63.54 ± 13.84	−0.16 ± 0.14	62.26 ± 14.19	63.09 ± 13.62	−0.06 ± 0.18	59.79 ± 13.29	59.81 ± 12.84	0.00 ± 0.14
Cross	21.33 ± 8.43	18.59 ± 20.76	0.19 ± 0.14	22.61 ± 9.32	17.57 ± 8.98	0.53 ± 0.17*	21.99 ± 10.03	16.52 ± 8.48	0.58 ± 0.14*
TB	3.29 ± 3.27	3.45 ± 3.20	−0.05 ± 0.14	2.55 ± 2.93	2.44 ± 2.66	0.04 ± 0.18	1.72 ± 1.95	1.81 ± 1.96	−0.04 ± 0.14
Dribble	10.13 ± 5.17	9.40 ± 4.37	0.15 ± 0.13	8.45 ± 4.09	8.33 ± 4.55	0.03 ± 0.18	7.98 ± 4.43	7.88 ± 4.28	0.02 ± 0.14
AS	54.59 ± 14.25	53.13 ± 13.07	0.11 ± 0.14	51.12 ± 13.94	48.89 ± 13.95	0.16 ± 0.18	46.90 ± 13.09	45.45 ± 14.23	0.10 ± 0.14
Corner	5.84 ± 2.87	4.85 ± 2.79	0.34 ± 0.14*	5.80 ± 3.10	4.63 ± 2.86	0.38 ± 0.17*	5.27 ± 3.15	4.13 ± 2.80	0.38 ± 0.14*
Offside	2.89 ± 2.31	2.82 ± 1.92	0.03 ± 0.14	2.78 ± 2.13	2.24 ± 2.02	0.26 ± 0.17*	2.73 ± 2.10	2.19 ± 1.90	0.27 ± 0.14*
Tackle	20.55 ± 6.09	20.18 ± 5.77	0.06 ± 0.14	20.19 ± 5.79	20.52 ± 5.79	−0.06 ± 0.18	19.57 ± 6.22	20.12 ± 5.95	−0.09 ± 0.14
Foul	12.80 ± 4.25	13.48 ± 4.51	−0.15 ± 0.14	13.18 ± 4.06	13.81 ± 4.42	−0.15 ± 0.18	13.67 ± 4.16	13.95 ± 4.69	−0.06 ± 0.14
YC	1.37 ± 1.13	1.92 ± 1.28	−0.45 ± 0.14*	1.76 ± 1.23	2.05 ± 1.10	−0.25 ± 0.17*	1.90 ± 1.34	2.17 ± 1.29	−0.20 ± 0.14*

*Descriptive statistics of teams in Tables 2–5 are presented as mean ± standard deviation (M ± SD). Effect sizes (ES) are presented as the magnitude of the true difference in means ± 90% confidence interval. Asterisks indicate that the p-value is lower than the significance level of 0.05. Abbreviations: ShotOT, shot on target; ShotOP, shot from open play; ShotSP, shot from set piece; ShotCA, shot from counter-attack; PS, pass success; SB, short ball; LB, long ball; TB, through ball; AS, aerial success; YC, yellow card.*

**TABLE 5:** Comparisons of technical match statistics between home and away teams considering match outcomes in the knockout stage of the UEFA Champions League.

Variable	Win			Draw			Lose		
	Home	Away	Effect size	Home	Away	Effect size	Home	Away	Effect size
	(M ± SD)	(M ± SD)	(ES ± 90%CI)	(M ± SD)	(M ± SD)	(ES ± 90%CI)	(M ± SD)	(M ± SD)	(ES ± 90%CI)
Shot	15.88 ± 5.79	13.97 ± 4.88	0.34 ± 0.26*	13.50 ± 7.10	11.10 ± 4.65	0.40 ± 0.36	12.86 ± 5.05	10.37 ± 5.21	0.47 ± 0.26*
ShotOT	6.50 ± 2.59	5.78 ± 2.50	0.28 ± 0.26	4.02 ± 2.10	4.05 ± 2.24	−0.01 ± 0.37	4.12 ± 2.23	3.27 ± 2.01	0.40 ± 0.26*
ShotOP	10.69 ± 4.78	9.60 ± 4.32	0.24 ± 0.26	9.17 ± 5.48	7.45 ± 3.66	0.36 ± 0.36	8.77 ± 3.88	6.94 ± 4.15	0.44 ± 0.26*
ShotSP	3.75 ± 2.31	2.67 ± 1.85	0.49 ± 0.24*	3.43 ± 2.50	2.55 ± 1.68	0.41 ± 0.36	3.35 ± 2.14	2.44 ± 1.75	0.47 ± 0.28*
ShotCA	0.56 ± 0.83	0.67 ± 0.99	−0.13 ± 0.26	0.24 ± 0.48	0.31 ± 0.60	−0.13 ± 0.36	0.00 ± 0.00	0.31 ± 0.56	−0.63 ± 0.17*
Possession	52.52 ± 13.57	53.31 ± 11.93	−0.06 ± 0.26	49.43 ± 13.87	50.57 ± 13.87	−0.08 ± 0.36	46.69 ± 11.93	47.48 ± 13.57	−0.06 ± 0.26
Pass	523.17 ± 154.54	555.76 ± 162.47	−0.21 ± 0.26	503.52 ± 146.65	513.98 ± 149.48	−0.07 ± 0.37	477.22 ± 113.48	470.85 ± 140.83	0.05 ± 0.24
PS	80.69 ± 8.09	82.52 ± 7.00	−0.24 ± 0.26	79.43 ± 6.70	79.26 ± 7.82	0.02 ± 0.37	79.97 ± 5.90	77.96 ± 8.31	0.26 ± 0.23
SB	439.76 ± 150.16	474.48 ± 160.37	−0.23 ± 0.26	418.79 ± 141.82	431.69 ± 139.79	−0.09 ± 0.36	397.86 ± 108.27	392.54 ± 135.44	0.04 ± 0.26
LB	59.73 ± 13.82	61.28 ± 13.64	−0.11 ± 0.26	58.81 ± 14.11	62.74 ± 17.11	−0.25 ± 0.36	55.95 ± 12.38	58.97 ± 13.61	−0.23 ± 0.26
Cross	19.92 ± 8.14	16.48 ± 7.78	0.42 ± 0.26*	22.74 ± 10.63	16.07 ± 8.32	0.66 ± 0.34*	20.95 ± 9.12	16.88 ± 8.03	0.47 ± 0.26*
TB	3.77 ± 4.32	3.52 ± 3.89	0.06 ± 0.26	3.60 ± 5.12	3.12 ± 2.93	0.11 ± 0.36	2.47 ± 2.22	2.47 ± 2.91	0.00 ± 0.26
Dribble	10.99 ± 5.41	10.43 ± 5.24	0.10 ± 0.26	11.40 ± 5.21	10.29 ± 4.31	0.23 ± 0.36	10.91 ± 5.41	8.84 ± 4.89	0.40 ± 0.26*
AS	52.39 ± 13.59	51.16 ± 12.11	0.09 ± 0.26	47.83 ± 14.32	52.17 ± 14.32	−0.30 ± 0.36	48.88 ± 12.11	47.64 ± 13.60	0.09 ± 0.26
Corner	5.68 ± 2.90	4.40 ± 2.43	0.45 ± 0.26*	5.19 ± 3.26	4.69 ± 2.87	0.16 ± 0.36	5.14 ± 2.85	4.23 ± 2.70	0.33 ± 0.26*
Offside	2.85 ± 2.19	2.83 ± 1.89	0.01 ± 0.26	2.26 ± 1.99	2.45 ± 1.85	−0.10 ± 0.36	2.00 ± 1.73	2.17 ± 1.99	−0.09 ± 0.26
Tackle	21.26 ± 6.46	21.22 ± 5.46	0.01 ± 0.26	20.81 ± 5.93	21.33 ± 6.73	−0.08 ± 0.36	19.50 ± 5.58	21.13 ± 6.27	−0.27 ± 0.26
Foul	13.52 ± 4.75	13.69 ± 4.95	−0.04 ± 0.26	13.24 ± 4.38	14.29 ± 5.06	−0.22 ± 0.36	13.33 ± 3.58	14.80 ± 4.98	−0.32 ± 0.23*
YC	1.93 ± 1.37	1.98 ± 1.37	−0.04 ± 0.26	1.86 ± 1.30	2.57 ± 1.50	−0.50 ± 0.35*	2.00 ± 1.11	2.65 ± 1.48	−0.46 ± 0.23*

*Descriptive statistics of teams in Tables 2–5 are presented as mean ± standard deviation (M ± SD). Effect sizes (ES) are presented as the magnitude of the true difference in means ± 90% confidence interval. Asterisks indicate that the p-value is lower than the significance level of 0.05. ShotOT, shot on target; ShotOP, shot from open play; ShotSP, shot from set piece; ShotCA, shot from counter-attack; PS, pass success; SB, short ball; LB, long ball; TB, through ball; AS, aerial success; YC, yellow card.*

## Discussion

The aim of this study was to identify the interactive effects of match location, match outcome, and competition stage regarding teams’ technical performance in the UEFA Champions League. First, a general comparison was made to identify the differences in the technical performance of teams between the group stage and the knockout stage. Then, further comparisons were conducted to examine the interactive effects among three competition situations on the technical performance of teams.

Results demonstrated that the teams’ technical performances did not differ much between the group and knockout stage, only the number of dribbles and yellow cards from the knockout stage were higher than the group stage. These findings may indicate that the matches teams played during the knockout stage were highly competitive compared to the group stage matches. Given the high importance of the knockout stage and the presence of higher-quality teams in this stage (Lidor et al., 2010), more aggressive defensive actions were committed to prevent the opponent from scoring a goal, and then more yellow cards given. Accordingly, when facing a team with balanced defense, especially in the knockout stage, it is difficult to generate unstability in a strong defensive line with ball possession by creating precise, well-constructed, and repeatable interactions among individuals (Chassy, 2013; Hewitt et al., 2016). In this case, creating one-against-one opportunities for the key players and using their individual ability (e.g., dribble) to break down the defensive line may increase the possibility of scoring a goal. However, a previous study by Yi et al. (2020b) found that these two variables had no significant differences between the group stage and the knockout stage for players from five playing positions in the UEFA Champions League. This disparity may be partly explained by the accumulative effect of players’ performances—that is, teams’ performance in a technical indicator includes all players’ performances during match play, and the accumulation of trivial differences in the players’ performances may result in significant differences at the team level.

In light of the limited effects of the competition stage on teams’ technical performances in isolation (Rein and Memmert, 2016), we hypothesized that novel insights about the match characteristics of teams between the group stage and the knockout stage could be obtained when evaluating teams’ technical performances across match locations, match outcomes, and competition stages. Our results confirmed the disparities between the group stage and the knockout stage by considering three match results and two match locations. Firstly, in the group stage, significant differences were found in the variables related to defense in the comparisons across three match results when played at home, while there were no significant differences in any variables related to defense when played away. However, the knockout stage was showed an opposite trend, where significant differences were found in the variables related to defense when played away, while no significant differences were found when played at home. These findings may due to the fact that the number of variables related to defense were increased when playing away compared to when playing at home in both competition stages, and the matches played in the knockout stage achieved higher number of variables related to defense than the group stage in both match locations. The reason for these changes could be easily explained by the high competitiveness of the two-stage knockout competition and the home advantage effect which have been well-discussed previously (Lago-Peñas and Lago-Ballesteros, 2011; Flores et al., 2015; Yi et al., 2020b). We also found that significant differences in more variables for the group stage than for the knockout stage regarding the differences in all three types of variables under the comparisons across three match results in both home and away matches, and the comparisons among two match locations in winning, drawing, and losing matches. This finding may indicate that the key performance indicators (KPIs) may differ between the group stage and the knockout stage (Yi et al., 2020b). The greater differences in the quality of teams in the group stage than the knockout stage may be a reason for the differences in the number of variables that showed significant differences among match outcomes. Further research is necessary to identify the KPIs for both sides and to inform ways in which these keys can be improved. Furthermore, in the group stage, significant differences were found between drawing and losing matches in most of the variables related to goal scoring and offense when playing at home and away, while no variables showed significant differences in the comparison of drawing and losing matches when playing at home and away in the knockout stage. This disparity may be partly due to the relatively smaller differences in the technical skills between teams that played in the knockout stage (Yi et al., 2020b), but the tactical and physical factors should also be considered in the interpretation of the non-significant technical differences between drawing and losing matches in the knockout stage. Additionally, in light of the non-significant differences between drawing and losing matches and their significant differences with winning matches in goal scoring and offense related variables, these findings may provide some insights for the identification of KPIs of winning a match in the knockout stage.

There is a common belief based on experiential knowledge that there are differences in the technical characteristics of teams between the group stage and knockout stage in the UEFA Champions League. Quantitative research has also been conducted by previous researchers attempting to quantify the differences, but the effects of situational variables were not accounted for Yi et al. (2020a,b). To the best of our knowledge, the present study is the first to interpret the interactive effects of situational variables on the technical performance of teams among competition stages. More detailed differences could be identified in order to provide more practical references for match preparation in different competition stages.

Moreover, KPIs can be examined more accurately by considering the interactive effects of situational variables. The current study identified that most of the variables related to goal scoring and offense (shot, shot on target, shot from open play, shot from counter-attack, possession, etc.) were the KPIs that could differentiate winning teams and non-winning teams across different competition scenarios, which is in line with the findings from previous studies (Lago-Ballesteros and Lago-Peñas, 2010; Lago-Peñas et al., 2010, Lago-Peñas and Lago-Ballesteros, 2011; Liu et al., 2015a). Specifically, goal scoring variables were identified as KPIs in both competition stages regardless of the competition contexts. It was emphasized that creating more scoring opportunities by employing suitable strategies and tactics, and improving the efficiency of shots would be a way for teams to succeed. Some variables related to offense (long ball, through ball, aerial success, and corner) that have previously been identified as the keys for successful teams (Liu et al., 2015b; Yi et al., 2019a) were also identified in the current study for winning teams in the group stage, but the same was not true for the knockout stage. These findings highlighted the importance of considering the competition stage in the identification of KPIs, and the relatively greater difference in the quality of teams in the group stage may once again account for the differences between competition stages. In the group stage, the stronger teams may more easily obtain superiority in goal scoring and offense and weaker teams tend to adopt a strategy of counter-attack. However, this superiority may be weakened in the more balanced matches in the knockout stage. Furthermore, new findings were also found in the variables related to defense; the yellow card showed significant differences between winning teams and non-winning teams when playing at home in the group stage, while no significant differences were found when playing away. It is noteworthy that the knockout stage presented the opposite trend to the group stage. It was suggested that the yellow card could be considered as a KPI during home matches played in the group stage and the away matches played in the knockout stage. This finding may provide more practical insights for coaches relative to the KPIs identified in previous studies without taking the situational variables of match location and competition stage into account.

The current study may also provide a holistic understanding of the differences in the technical performance between home and away matches, and the results presented are consistent with previous research (Lago-Penas et al., 2011; Liu et al., 2015b). Generally, teams obtained higher values in variables related to goal scoring and offense, and lower values in variables related to defense in home matches than in away matches among competition situations. More importantly, our results may support and clarify a notion that the indicators of home advantage are not completely equivalent to the KPIs of teams’ match performances, which is unclear in the available literature. Some variables showed significant differences between home and away matches and could be identified as the key indicators of home advantage, but they were not the KPIs that could distinguish winning and non-winning teams. For example, the current study identified that corner is an important indicator of home advantage; the number of corners from the home matches was higher than those from the away matches in most of the pairwise comparisons among situational variables. However, the number of corners from the winning teams was not significantly higher than those from the non-winning teams. Moreover, the variable of cross has previously been identified as a KPI of teams’ match performance (Liu et al., 2016b), but it was neither a KPI nor a predictor of home advantage in this study.

## Conclusion

The current study investigated the interactive effects of situational variables on the technical performance of teams in the UEFA Champions League. The consideration of interaction among competition stage, match location, and match outcome provided more detailed and practical insights regarding the differences between the group stage and the knockout stage, and in the identification of KPIs of match performance as well as the indicators of home advantage. The variables that could differentiate the winning and non-winning matches, and the home and away matches, were different across competition contexts. Therefore, more detailed differences among competition scenarios were revealed, and these would not have been detected in analyses conducting the comparisons in isolation. The competition contexts had greater impacts on the technical performance of teams in the group stage than the knockout stage. The importance of the variables related to goal scoring was also confirmed in this study, and the significant differences could be found in most of the pairwise comparisons. Moreover, the variables related to goal scoring can better differentiate the technical performance between home and away matches in different match outcomes than other two types of variables, except for drawing matches in the knockout stage.

Our findings may help teams to prepare for upcoming match playing in specific competition situations, assessing their own or their opponent’s weaknesses and strengths. The identified KPIs under different situations can be elaborated in the training session or evaluated during the post-match assessment to optimize the team’s performance. In addition, in the light of the inconsistencies between KPIs and the indicators of home advantage, both factors should be interpreted with caution in future research. However, some limitations should be noted. Firstly, only technical variables were examined, and physical variables were not accounted for. Secondly, future research should consider the quality of teams to establish more holistic performance profiles.

## Data Availability Statement

The raw data supporting the conclusions of this article will be made available by the authors, without undue reservation.

## Ethics Statement

The studies involving human participants were reviewed and approved by Shanghai University of Sport, 200438 Shanghai, China. Written informed consent from the participants was not required to participate in this study in accordance with the national legislation and the institutional requirements.

## Author Contributions

QY, XW, and YG: conceptualization. QY: methodology, software, data curation, and visualization. JY, XW, and YG: validation. QY: formal analysis and writing—original draft preparation. JY, XW, YG, and M-ÁG-R: writing—review and editing. XW, YG, and M-ÁG-R: supervision. XW: funding acquisition. All authors have read and agreed to the published version of the manuscript.

## Conflict of Interest

The authors declare that the research was conducted in the absence of any commercial or financial relationships that could be construed as a potential conflict of interest.

## Publisher’s Note

All claims expressed in this article are solely those of the authors and do not necessarily represent those of their affiliated organizations, or those of the publisher, the editors and the reviewers. Any product that may be evaluated in this article, or claim that may be made by its manufacturer, is not guaranteed or endorsed by the publisher.

## Footnotes

1
http://www.whoscored.com
